# Efficacy and safety of radiofrequency ablation versus surgical sympathectomy in palmar hyperhidrosis

**DOI:** 10.1038/s41598-024-57834-0

**Published:** 2024-04-01

**Authors:** Yiyue Zhong, Yanwen Zhu, Jiayan Li, Xiaowei Yang, Zhiying Feng, Haipeng Liu, Zhu Liang, Baoquan Lin, Zhifeng Liu, Xin Wang, Weibin Luo, Jian Zhu, Bin Li, Shangdao Lai, Weize Jiang, Jiayuan Wu, Daheng Li, Liangqing Zhang, Bing Huang, Jing Tang

**Affiliations:** 1 Department of Anaesthesiology and Pain Medicine, Affiliated Hospital of Guangdong Medical University, No. 57 People Avenue South, Zhanjiang, 524001 Guangdong China; 2 Department of Thoracic Cardiovascular Surgery, Gaozhou People’s Hospital, No. 89 Xiguan Road, Gaozhou, 525200 Guangdong China; 3 Department of Thoracic Cardiovascular Surgery, The First Hospital of Wuhan, No. 215 Zhongshan Road, Qiaokou District, Wuhan, 430070 China; 4 Department of Pain Medicine, The First Affiliated Hospital of Zhejiang University, Hangzhou, 310003 China; 5 Department of Pain Medicine, Gansu Provincial Hospital, No. 204, Donggang West Road, Lanzhou, 730000 Gansu China; 6 Department of Thoracic Cardiovascular Surgery, Affiliated Hospital of Guangdong Medical University, No. 57 People Avenue South, Zhanjiang, 524001 Guangdong China; 7 Department of Thoracic Cardiovascular Surgery, The 900Th Hospital of Joint Logistic Support Force, No. 156 West Second Ring North Road, Fuzhou, 350000 Fujian China; 8 Department of Critical Care Medicine, General Hospital of Southern Theatre Command of PLA, Guangzhou, 510010 China; 9 Department of Pain Medicine, The Third People’s Hospital of Huizhou, No. 1, Qiaodong Xuexiu Street, Huicheng District, Huizhou, 516000 Guangdong China; 10 Department of Thoracic Cardiovascular Surgery, The Second People’s Hospital of Shenzhen, No. 3002 Sungang West Road, Futian District, Shenzhen, 518000 Guangdong China; 11 Department of Thoracic Cardiovascular Surgery, General Hospital of Central Theater Command of the People’s Liberation Army, 627#, Wuluo Road, Wuchang District, Wuhan, 430070 Hubei China; 12 Department of Thoracic Cardiovascular Surgery, Shanghai Chest Hospital, Shanghai Jiao Tong University, No. 241 West Huaihai Road, Shanghai, 310000 China; 13 Department of Pain Medicine, Meizhou People’s Hospital (Huangtang Hospital), Meizhou Hospital Affiliated to Sun Yat-Sen University, No. 63 Huangtang Road, Meijiang District, Meizhou, 514031 China; 14 Department of Pain Medicine, China Railway Fuyang Central Hospital, No. 161 Xingfu Road, Yingdong District, Fuyang, 236000 Anhui China; 15 Department of Clinical Research, Affiliated Hospital of Guangdong Medical University, No. 57, South of People Avenue, Zhanjiang, 524001 Guangdong China; 16 Department of Anaesthesiology and Pain Center, The Affiliated Hospital of Jiaxing University, Jiaxing, 314000 Zhejiang China

**Keywords:** Palmar hyperhidrosis, Radiofrequency ablation, Video-assisted thoracoscopic sympathectomy, Efficacy, Safety, Neuroscience, Neurology, Skin diseases, Quality of life, Outcomes research

## Abstract

Radiofrequency ablation (RFA) comparative efficacy of treatments using video-assisted thoracoscopic sympathectomy (VATS) in the long term remains uncertain in patients with palmar hyperhidrosis (PHH). This study aimed to compare the efficacy and safety of RFA and VATS in patients with PHH. We recruited patients aged ≥ 14 years with diagnosed PHH from 14 centres in China. The treatment options of RFA or VATS were assigned to two cohort in patients with PHH. The primary outcome was the efficacy at 1-year. A total of 807 patients were enrolled. After propensity score matching, the rate of complete remission was lower in RFA group than VATS group (95% CI 0.21–0.57; *p* < 0.001). However, the rates of palmar dryness (95% CI 0.38–0.92; *p* = 0.020), postoperative pain (95% CI 0.13–0.33; *p* < 0.001), and surgery-related complications (95% CI 0.19–0.85; *p* = 0.020) were lower in RFA group than in VATS group, but skin temperature rise was more common in RFA group (95% CI 1.84–3.58; *p* < 0.001). RFA had a lower success rate than VATS for the complete remission of PHH. However, the symptom burden and cost are lower in patients undergoing RFA compared to those undergoing VATS.

Trial Registration: ChiCTR2000039576, URL: http://www.chictr.org.cn/index.aspx.

## Introduction

Hyperhidrosis refers to sweating exceeding physiological needs and is considered a disease of the autonomic nervous system, with an unclear specific pathogenesis^1–3^. Two previous studies reported a prevalence of 2.8% in the United States and 2.08% in China, w corresponding to 7.8 million American and 29.1 million Chinese individuals with hyperhidrosis^4,5^. The main symptomatic parts include the hand, axilla, craniofacial region, and feet^4,5^. Hyperhidrosis substantially affects patients' social life and work and may even cause depression in severe cases; severely affected patients have skin maceration and secondary microbial infections^6,7^.

Regarding hyperhidrosis treatment, the options are surgical and nonsurgical^8,9^. Nonsurgical treatment includes direct injections or through iontophoresis of botulinum toxin^6,10^, topical antiperspirants (aluminium chloride hexahydrate)^11,12^, laser treatment^13^, and oral medications (anticholinergics, beta-blockers, and benzodiazepines)^9^, and these treatments have limitations and a higher rate of recurrence. Therefore, surgical sympathectomy may be considered a final option when more conservative treatments have failed^9^. Video-assisted thoracoscopic sympathectomy (VATS) is the most commonly used surgical option to treat palmar hyperhidrosis (PHH)^11^. However, it requires general anaesthesia and has more trauma than other options^14,15^, and more compensatory hyperhidrosis occurs after VATS^9^, leading to many challenges for treatment in those patients.

As a minimally invasive procedure, radiofrequency ablation (RFA) has many satisfactory advantages, such as minor trauma and quick recovery^16^. The therapeutic mechanism of RFA is a thermal effect for tissue coagulation without causing neuromuscular excitation or pain^17^. Presently, RFA has been widely used to treat tumours and chronic pain, has achieved remarkable results^18^, and is effective for hyperhidrosis treatment^9^. However, to date, only two studies have been designed to compare the effectiveness of RFA and VATS for hyperhidrosis treatment^8,19^. One study reported that RFA has long-term patient satisfaction, with a success rate of 75% for treating palmar hyperhidrosis (PHH) in 46 patients^8^. Another nonrandomized controlled clinical trial supports the view of surgical sympathectomy as the gold-standard treatment in severe cases of PHH in 31 patients^19^. These studies with limited clinical data have not provided strong evidence for the management of hyperhidrosis^11^.

Here, we conducted a nationwide multicentre cohort study to compare the efficacy and safety of RFA and VATS in patients with PHH to improve clinical practice.

## Methods

### Study design

The comparative efficacy research to assess the use of RFA and VATS for PHH was an investigator-initiated, prospectively collected data, retrospective controlled cohort study done at 14 centers in China between 2015 and 2019 (Supplementary Statistical Analysis Plan).

### Study participants

Eligible participants were aged 14 years or older, diagnosed with primary PHH and treated with RFA or VATS. Patients who had non-palmar hyperhidrosis and non-interventional treatment for hyperhidrosis were excluded^8^. The study was approved by the institutional ethics committees at the principal investigator centre on September 16, 2020, and was being recognized at 13 other centres. This study was registered with the China Clinical Trials Registry on November 1, 2020 (ChiCTR2000039576). All the patients provided written informed consent before RFA or VATS for PHH treatment, but they were not invited to be included in this study at that time. Therefore, all data were obtained after ethical approval, and patients were explained by our follow-up that they were included in this study, the ethics approve it was exempted from signing a written informed consent form. The study was performed according to the Declaration of Helsinki and Good Clinical Practice principles.

### Study cohort

**Figure 1:**
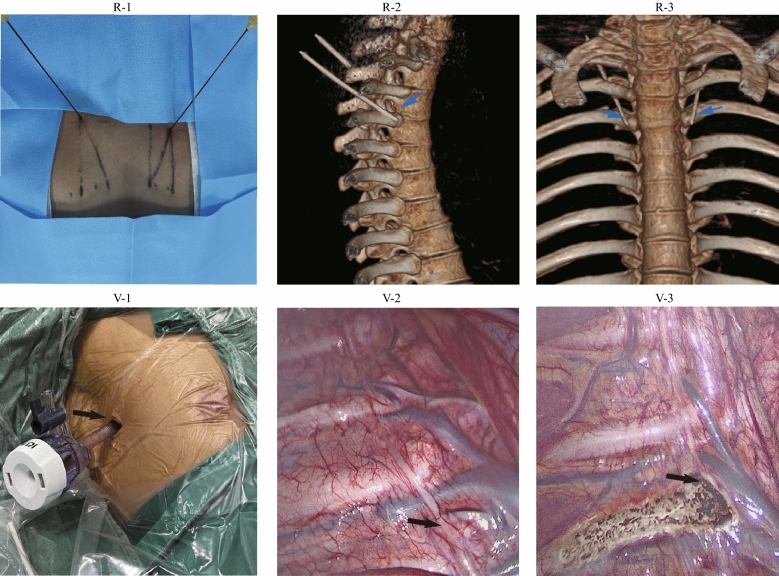
Radiofrequency ablation (RFA) and video-assisted thoracoscopic sympathectomy (VATS) for treatment, with pathways and procedures at the fourth thoracic level. R-1: Body surface for the operative approach in RFA. R-2: Lateral film for 3-dimensional X-ray computed tomography imaging in RFA. R-3: Positive film for 3-dimensional X-ray computed tomography imaging in RFA. V-1: Body surface for the operative approach in VATS (third/fourth intercostal space). V-2: Straight view of the sympathetic nerve before surgery with VATS. V-3: Straight view of the sympathetic nerve after surgery with VATS.

The treatment options were determined according to a prespecified analysis plan between the RFA and VATS frameworks (Fig. 1).

Patients in this study cohort included two distinct groups: the RFA group, which comprised patients who underwent RFA applied unilaterally; the control (VATS) group, which comprised patients who underwent electrosurgery sympathectomy (bilateral of Thoracic 4 and or Thoracic 3 ganglia according to the actual condition) using video-assisted thoracoscopy under general anaesthesia with double-lumen endotracheal intubation^8,11^. In the RFA group, RFA was performed for PHH according to local practice at the study centres because no authoritative practical guidelines were available. First, the patients whether underwent a homogeneity technique protocol at the participating centres by judge of surgeon or operator. Following subcutaneous local anaesthetic infiltration, the 5 mm active cannula of the RFA device was advanced to the bilateral of thoracic 4 sympathetic ganglions under fluoroscopic guidance (X-ray computed tomography). When the probe reached the desired point, the level of the cannula was tested by 3-dimensional image reconstruction. After neurophysiological testing^8^, RFA thermal coagulation was applied at 90 °C for 180 s, and 2 ml of 2% lidocaine was spread through the cannula after thermal coagulation was necessary. The decision to treat PHH was not determined by study design but instead by the physician and patient and might be influenced by regional health policy practices (Social Security fund policy and Medical Insurance Policy).

### Variables

Control variables, including demographic data, clinical disease and family history, were collected preoperatively from the medical charts at each participating centre. The variables of the Hyperhidrosis Disease Severity Scale (HDSS)^1^ and the quality of life (QOL) questionnaire^19^ by follow-up to be evaluated between pre-operation and post-operation, respectively. The HDSS questionnaire comprises four statements, each receiving a score of 1–4, with 1 being the mildest grade and 4 the worst^20^. The QOL questionnaire comprises twenty statements, each receiving a score of 1–5, with 20 being the mildest grade and 100 the worst. The outcome variables included the clinical efficacy, safety (intraoperative and postoperative complications), patient satisfaction, HDSS and QOL questionnaire, compensatory hyperhidrosis, and symptom recurrence follow-up data at postoperative months 1, 3, 6, and 12 (1 year), as previously reported^8,21,22^. For patient satisfaction, the chief complaint of patients was made by phone call and return follow-up to be evaluated. To evaluate postoperative QOL, the final data of the follow-up questionnaire was used. All follow-up was conducted by an external blinded endpoint committee, established at the principal investigator's institution, to ensure unbiased assessments. For preoperative assessment, clinical data were obtained from medical records and medical documents. Postoperatively, the follow-up was carried out by investigators who had undergone standardized training, thus guaranteeing the reliability and consistency of data collection and analysis.

### Outcomes measures

The primary outcome was the clinical efficacy at 1 year. Successful clinical efficacy was defined as complete remission after treatment for PHH, and ineffective treatment was defined as no remission or a few partial remissions after treatment for PHH. Surgical failure was defined as no improvement in hyperhidrosis symptoms postoperatively. The HDSS and QOL were assessed preoperatively and postoperatively as components of the primary outcome. The secondary outcomes included symptom recurrence, complications, compensatory hyperhidrosis, patient satisfaction, length of stay, and hospital costs.

### Sample size calculation

The study sample included patients aged ≥ 14 years who had received RFA or VATS for PPH, assuming an efficacy rate of 68–100% with VATS according to published literature^11^. The power analysis for the equivalence tests of two independent proportions was performed using PASS 11 software (NCSS, LLC., Kaysville, Utah, USA), which Sample sizes of 150 in the treatment group and 150 in the control group achieved 90% power to detect equivalence. The margin of equivalence, given in terms of the difference, extended from 0.20 to 0.20. The calculations assume that two, one-sided Z tests are used. The significance level is Alpha targeted at 0.05.

### Statistical analysis

The RFA and VATS cohorts were analysed separately for HDSS and QOL preoperatively and postoperatively. Patients who were lost to follow-up were excluded from the analysis. In patients with PHH, the baseline characteristics were compared between patients receiving RFA and VATS using standardized mean differences (SMDs). The propensity score for receiving RFA was estimated using a logistic regression model. Covariates included in the model were demographics (age and sex), family history, HDSS and QOL preoperatively. Propensity-score matching was implemented using a nearest-neighbour strategy, with a minimum caliper of 0.1^23^. The ratio was one patient receiving RFA matched with one patient receiving VATS. The SMD was used to assess the balance of baseline covariates between the RFA and VATS groups in the matched cohort. An SMD of less than 0.10 indicated a good balance^24^. In the matched cohort, the nonnormally distributed length of stay and hospitalization costs were converted to categorical variables based on the median. The primary and secondary outcomes to compare the distributions of categorical variables using chi-squared test in the unmatched cohort and logistic regression models in the matched cohort are reported with odds ratios (ORs) and 95% confidence intervals (CIs) for the two treatment groups. To test whether the findings of the patient-level analysis might be due to a causal effect of RFA, we used a further adjusted time-varying Cox proportional hazard model to estimate the hazard ratio for ineffective treatment in the matched cohort (Statistical Analysis Plan in the Supplementary Appendix, Fig. 1).

Statistical analyses were performed using R 3.6.3 software.

### Ethics approval

This study was approved by the institutional review board (IRB) of Affiliated Hospital of Guangdong Medical University (No. PJ2020-079). Data from the surgical options cohort do not involve any personally identifiable data. Thus, the IRB approved the cohort study without informed consent from participants.

### Transparency statement

The manuscripts guarantors (all authors) affirm that the manuscript is an honest, accurate, and transparent account of the study being reported; that no important aspects of the study have been omitted; and any discrepancies from the study as planned have been explained. This is an Open Access article distributed in accordance with the terms of the Creative Commons Attribution (CC BY 4.0) license, which permits others to distribute, remix, adapt and build upon this work, for commercial use, provided the original work is properly cited. See: http://creativecommons.org/licenses/by/4.0/.

## Results

### Baseline characteristics

**Figure 2:**
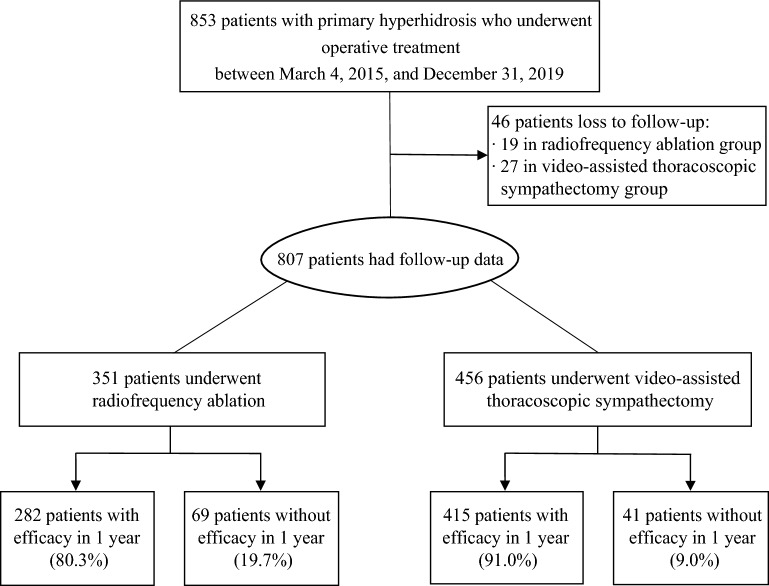
Study profile of the selection of study participants.

Between March 4, 2015, and December 31, 2019, at 14 centres in China**,** 853 patients were enrolled, and 46 patients were excluded because they were lost to follow-up. Thus, the population for our study of the clinical efficacy included 807 patients: 351 in the RFA group and 456 in the VATS group (Fig. 2, Table S1 in the Supplementary Appendix). The median follow-up was 1.5 years (IQR 1.4–1.7) in the RFA group and 1.7 (IQR 1.4–2.3) in the VATS group. 19 (5.4%) patients in the RFA group and 27 (5.9%) in the control group were lost to follow-up. Because the proportion of patients with missing items was moderate, complete case analyses were performed. In the baseline cohort, although patients in the RFA and VATS groups were well matched for disease severity (HDSS), compared with the VATS group, patients who underwent RFA were older (mean age 25.1 years (SD 6.7) versus 23.0 years (SD 5.7); SMD 34.5%), more female (187/351 [53.3%] versus 215/456 [47.2%]; SMD 12.3%), and had lower QOL scores (SMD 19.1%) (Table 1). After propensity score matching, all the baseline characteristics of patients who received RFA and VATS were well balanced, with an SMD less than 0.10 (Table 1).

**Table 1:** Baseline characteristics, according to treatment with radiofrequency ablation (RFA) and video-assisted thoracoscopic sympathectomy (VATS), in propensity-score–matched patients with primary hyperhidrosis.

	Unmatched			Matched		
	RFA (n = 351)	VATS (n = 456)	SMD	RFA (n = 312)	VATS (n = 312)	SMD
Age, mean (SD), year	25.13 (6.69)	22.99 (5.65)	0.345	24.16 (5.94)	24.45 (6.18)	0.048
Female, sex, n (%)	187 (53.3)	215 (47.1)	0.123	175 (56.1)	164 (52.6)	0.071
Family history, n (%)	141 (40.2)	195 (42.8)	0.053	122 (39.1)	129 (41.3)	0.046
HDSS in preoperative, n (%)*			0.174			0.075
1	0 (0.0)	0 (0.0)		0 (0.0)	0 (0.0)	
2	26 (7.4)	16 (3.5)		14 (4.5)	19 (6.1)	
3	161 (45.9)	223 (48.9)		143 (45.8)	144 (46.2)	
4	164 (46.7)	217 (47.6)		155 (49.7)	149 (47.8)	
QOL in preoperative, n (%)			0.113			0.011
20–35	0 (0.0)	0 (0.0)		0 (0.0)	0 (0.0)	
36–51	0 (0.0)	0 (0.0)		0 (0.0)	0 (0.0)	
52–68	38 (10.8)	41 (9.0)		33 (10.6)	34 (10.9)	
69–84	174 (49.6)	210 (46.1)		153 (49.0)	152 (48.7)	
85–100	139 (39.6)	205 (45.0)		126 (40.4)	126 (40.4)	

*VATS* Video-assisted thoracoscopic sympathectomy, *RFA* Radiofrequency ablation, *HDSS* Hyperhidrosis Disease Severity Scale, *QOL* Quality of life questionnaire, *SMD* Standardized mean differences.

*1: My sweating is never noticeable and does not interfere with my daily activities; 2: My sweating is tolerable but sometimes it interferes with my daily activities; 3: My sweating is barely tolerable and frequently interferes with my daily activities; 4: My sweating is intolerable and always interferes with my daily activities.

### Primary outcome

**Figure 3:**
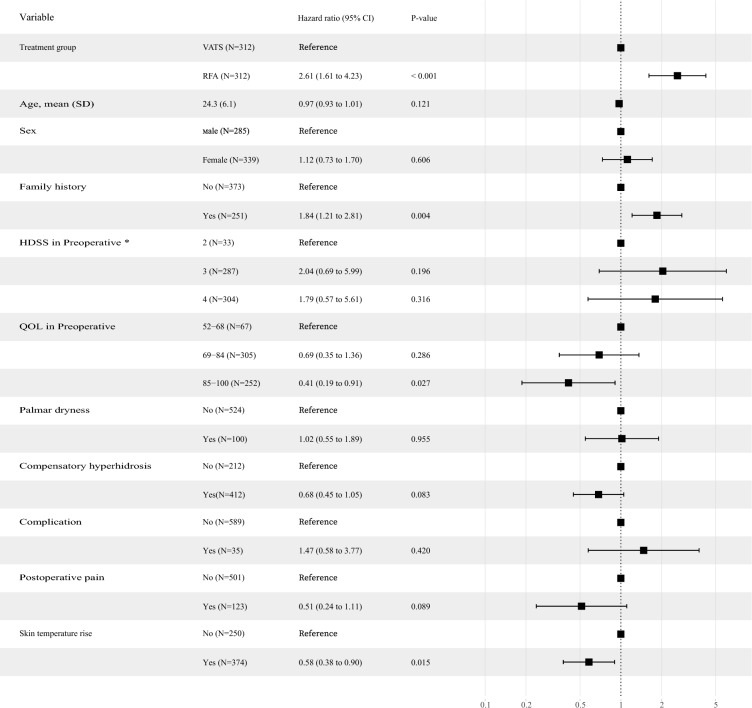
Multivariable models to estimate the hazard ratio of symptom recurrence using a time-varying Cox proportional hazard model. *1: My sweating is never noticeable and does not interfere with my daily activities; 2: My sweating is tolerable, but sometimes it interferes with my daily activities; 3: My sweating is barely tolerable and frequently interferes with my daily activities; 4: My sweating is intolerable and always interferes with my daily activities. *RFA* Radiofrequency ablation, *VATS* Video-assisted thoracoscopic sympathectomy, *HDSS* Hyperhidrosis Disease Severity Scale, *QOL* Quality of life questionnaire.

The postoperative association between treatment options and outcomes is presented in Table 2. In the matched cohort of 312 patients who underwent RFA and 312 patients who underwent VATS, the surgical failure rate was not significantly different between the RFA and VATS groups at the hospital (0.3% [1/312] versus 1.0% [3/312]; OR 2.84; 95% CI 0.26 to 72.59; *p* = 0.428). However, the rate of complete remission was 79.2% (247/312) to treat PHH in the RFA group and 91.3% (285/312) in the VATS group (OR 0.35; 95% CI 0.21 to 0.57; *p* < 0.001) at 1-year postoperatively. The multivariable Cox model showed that the risk of ineffective treatment was higher in the RFA group than in the VATS group (HR 2.61; 95% CI 1.61 to 4.23; *p* < 0.001) and higher in the group with a family history than in the group without a family history (HR 1.84; 95% CI 1.21 to 2.81; *p* = 0.004). However, the risk of ineffective treatment was lower with a worse quality of life (QOL > 84) than without (HR 0.41; 95% CI 0.19 to 0.91; *p* = 0.027) and with a skin temperature rise than without (HR 0.58; 95% CI 0.38 to 0.90; *p* = 0.014) (Fig. 3).

**Table 2:** Primary and secondary outcomes according to treatment with radiofrequency ablation (RFA) and video-assisted thoracoscopic sympathectomy (VATS), in propensity-score–matched patients with palmar hyperhidrosis.

	All patients (n = 807)	RFA (n = 351)	VATS (n = 456)	*x*^2^/t	Odds ratio (95% CI)	*p* value
Primary outcomes						
Clinical efficacy at hospital discharge, n (%)						
Surgical failure	9 (1.1)	5 (1.4)	4 (0.9)	0.539	1.624 (0.439–6.003)	0.513
Partial remission	156 (19.3)	65 (18.5)	91 (20.0)	0.263	0.928 (0.697–1.236)	0.653
Complete remission	642 (79.6)	281 (80.1)	361 (79.2)	0.097	1.011 (0.943–1.085)	0.792
Clinical efficacy at 1 year postoperatively, n (%)	697 (86.4)	282 (80.3)	415 (91.0)	19.17	0.404 (0.267–0.611)	< 0.001
HDSS in postoperative, n (%)*				14.757	0.745 (0.612–0.907)	0.003
1	686 (85.0)	280 (79.8)	406 (89.0)			
2	52 (6.4)	31 (8.8)	21 (4.6)			
3	39 (4.8)	25 (7.1)	14 (3.1)			
4	30 (3.7)	15 (4.3)	15 (3.3)			
QOL in postoperative, n (%)				18.047	0.676 (0.556–0.823)	< 0.001
20–35	615 (76.2)	248 (70.7)	367 (80.5)			
36–51	114 (14.1)	53 (15.1)	61 (13.4)			
52–68	62 (7.7)	38 (10.8)	24 (5.3)			
69–84	11 (1.4)	9 (2.6)	2 (0.4)			
85–100	5 (0.6)	3 (0.9)	2 (0.4)			
Secondary outcomes						
Symptom recurrence, n (%)	101 (12.5)	64 (18.2)	37 (8.1)	18.55	2.247 (1.537–3.286)	< 0.001
Palm dry in postoperative, n (%)		43 (12.3)	84 (18.4)	5.694	0.665 (0.473–0.934)	0.019
Compensatory hyperhidrosis, n (%)	523 (64.8)	235 (67.0)	288 (63.2)	1.252	1.060 (0.958–1.173)	0.266
Patient satisfaction, n (%)				3.242	1.055 (0.890–1.251)	0.539
Dissatisfaction	29 (3.6)	16 (4.6)	13 (2.9)			
Moderate	90 (11.2)	42 (12.0)	48 (10.5)			
Satisfaction	264 (32.7)	106 (30.2)	158 (34.6)			
Complete satisfaction	424 (29.4)	187 (53.3)	237 (52.0)			
Complication, n (%)	50 (6.2)	13 (3.7)	37 (8.1)	32.789	1.204 (1.071–1.353)	0.001
Dyspnea	1 (0.1)	1 (0.3)	0 (0.0)			
Axillary pain	1 (0.1)	1 (0.3)	0 (0.0)			
Acute chest syndrome	3 (0.4)	3 (0.9)	0 (0.0)			
Pneumothorax	19 (2.4)	3 (0.9)	16 (3.5)			
Bradycardia	1 (0.1)	1 (0.3)	0 (0.0)			
Waist discomfort	3 (0.4)	3 (0.9)	0 (0.0)			
Incisional pain	8 (1.0)	0 (0.0)	8 (1.8)			
Incisional paralysis	1 (0.1)	0 (0.0)	1 (0.2)			
Shoulder-back pain	3 (0.4)	1 (0.3)	2 (0.4)			
Subcutaneous emphysema	1 (0.1)	0 (0.0)	1 (0.2)			
Pleural effusion	9 (1.1)	0 (0.0)	9 (2.0)			
Pain after discharge, (%)	166 (20.6)	32 (9.1)	134 (29.4)	49.871	0.310 (0.217–0.445)	< 0.001
Palm temperature rise, n (%)	473 (58.6)	253 (72.1)	220 (48.2)	46.445	1.494 (1.331–1.676)	< 0.001
Length of stay, mean (SD)	2.90 (1.58)	2.43 (1.22)	3.26 (1.72)	8.015	0.108 (0.617–1.032)	< 0.001
Hospital costs, mean (SD)	1739.6 (4949.7)	1013.9 (376.6)	2298.1 (6524.7)	4.194	306.2 (682.4–1885.9)	< 0.001

*VATS* Video-assisted thoracoscopic sympathectomy, *RFA* Radiofrequency ablation, *HDSS* Hyperhidrosis Disease Severity Scale, *QOL* Quality of life questionnaire, *SMD* Standardized mean differences.

*1: My sweating is never noticeable and does not interfere with my daily activities; 2: My sweating is tolerable but sometimes it interferes with my daily activities; 3: My sweating is barely tolerable and frequently interferes with my daily activities; 4: My sweating is intolerable and always interferes with my daily activities.

As a primary outcome component, the HDSS showed a significant reduction in the two groups preoperatively and postoperatively (*p* < 0.001), and quality of life (QOL questionnaire) was significantly improved in the two groups (*p* < 0.0001) (Table S2 in the Supplementary Appendix). However, compared with the RFA group, the postoperative HDSS (1) assessment improved more significantly in the VATS group at the end of follow-up (275/312 [88.1%] versus 245/312[78.5%]; OR 0.46; 95% CI 0.29 to 0.73; *p* = 0.001). Similarly, the postoperative QOL questionnaire (20–35) also improved more significantly in the VATS group (251/312[80.4%] versus 217/312[69.6%]; OR 0.54; 95% CI 0.37 to 0.78; *p* = 0.001) (Table 2).

### Secondary outcome

In the matched cohort, the number of patients with PHH who underwent RFA was higher than that who underwent VATS regarding symptom recurrence (OR 2.78, 95% CI 1.71 to 4.63; *p* < 0.001). By contrast, the rates of clinical symptoms after treatment showed that the rates of palmar dryness, postoperative pain, and surgery-related complications were lower in the RFA group than in the VATS group, but the rates of skin temperature rise were higher in the RFA group (Table 2). The common complications of VATS were pneumothorax (11/312 [3.5%]), pleural effusion (6/312 [1.9%]), and incisional pain (4/312 [1.3%]) (Table S1 in the Supplementary Appendix). The length of stay was shorter in the RFA group than in the VATS group (OR 0.21; 95% CI 0.13 to 0.33; *p* < 0.001). The hospital costs were lower in the RFA group than in the VATS group (OR 0.01; 95% CI 0.00 to 0.01; *p* < 0.001). Regarding compensatory hyperhidrosis and the chief complaint of patient satisfaction, we found no significant differences between RFA and VATS (Table 2).

## Discussion

Both RFA and VATS are considered successful procedures for PHH to complete remission. However, RFA (79.3%), compared with VATS (91.2%), resulted in a lower rate of complete remission for PHH 1 year postoperatively. The reason may be that the operator excises the sympathetic nerve more precisely under direct vision on the screen through video-assisted thoracoscopy^25^ and not just because the operator has relatively less experience eliciting this block^8^.

Surgery-related complications were less frequent in the two groups, a finding consistent with that in several previous studies demonstrating that the destruction of these nerves is safe and effective to stop hyperhidrosis of the palmar^9,11,14^. Complications have been described regarding two aspects. On the one hand, complications related to performing surgical procedures commonly occur during the perioperative period, such as dyspnoea, acute chest syndrome, incisional pain, pneumothorax, and pleural effusion. Additionally, complications can be related to excision of the sympathetic nerve, commonly appearing after hospital discharge, such as axillary pain, shoulder-back pain, compensatory hyperhidrosis, bradycardia, nasal obstruction, nerve injury, and intercostal neuralgia^21^. In this large sample cohort study, although not all complications were observed, they were considered tolerable because of the relative merits of improved hyperhidrosis symptoms, an improved quality of life and patient satisfaction in each patient with chief complaints.

In the present study, the advantages of RFA were its minimal invasiveness^16^, low postoperative painVB^17^, short length of stay, and low hospital cost. These advantages are reasonable and deserve positive recognition; RFA was used as a lesion generator for thermal ablation under fluoroscopic guidance using a 5 mm active cannula^8^. A more minimally invasive procedure that does not require general anaesthesia and greater trauma procedures^14,15,26^ leads to less postoperative pain^17^, possibly due to the unnecessary longer length of stay and reduced hospitalization costs for RFA procedures than for VATS procedures. Additionally, sympathetic vasoconstrictor reflexes after the destruction of the sympathetic ganglion in two group^27^, there is an increase in skin temperature in the affected areas of the patient. Interestingly, we found a higher rate of skin temperature rise in the RFA group than in the VATS group. This can be attributed to two potential factors. Firstly, the application of thermocoagulation at 90 °C for 180 s may exert a more extensive influence on the RFA of peripheral nerve tissue^8^. Secondly, VATS group with general anaesthesia greatly impairs thermoregulation and synchronously reduces the thresholds for vasoconstriction^28^. Our data showed a negative correlation between palmar temperature rise and inefficacy treatment. However, palmar temperature changes may not be used to predict a cure or guide surgical approaches^29^. In general, RFA options lead to a lower symptomatic burden, which is more likely to be accepted by patients, and more treatment options (one more RFA, or VATS) will be available in patients who undergo RFA when symptoms recur^22^.

In comparison, the advantage of VATS treatment for hyperhidrosis is that the sympathetic nerve can be excised more thoroughly, leading to more significant improvement in the symptoms of hyperhidrosis and quality of life, and the rate of symptom recurrence is lower in VATS than in RFA. A pathogenesis study of PHH showed that the cholinergic receptor nicotinic alpha 1 subunit and activin a receptor type 1 may be involved in the pathogenesis of primary hyperhidrosis^30,31^. Another genome-wide analysis of families with PHH showed that variants or mutations located outside the coding regions may be involved in the molecular pathogenesis of PHH^3^. The Cox proportional hazard model for ineffective treatment showed a higher risk in patients with a family history than in those without, likely because of variants or mutations located outside the coding regions^5,30^.

This study has several limitations. First, an observational study evaluating the clinical efficacy of RFA and VATS is potentially subjected to selection bias^32^. Although balance was achieved in each cohort by propensity score matching, patients selected for RFA may still differ regarding treatment history compared with patients receiving VATS, and seven centres did not perform RFA for PHH. However, sympathectomy is the final option of treatment when conservative treatments fail or are intolerable^11^, and the treatment history does not affect the clinical outcome of sympathectomy^3,30^. Second, the proposed different follow-up times were observed for the RFA or VATS treatment effect with HDSS and QOL must be independently assessed^22^. In this context, the endpoint assessment for the treatment of clinical efficacy for long-term outcomes might require increased attention^9^. Third, we acknowledge that the reliability of the measures should be interpreted cautiously due to the possible operator technical skills variation in this study^33^. Finally, missing data likely influenced the results, and we are not sure whether the inclusion of lost to follow-up data will affect the final analysis results of this study.

## Conclusions

This study suggests that performing RFA had a lower success rate than VATS for the complete remission of palmar hyperhidrosis. However, the symptom burden and cost are lower in patients undergoing RFA compared to those undergoing VATS.

### Supplementary Information

Supplementary Information 1.

Supplementary Information 2.

Supplementary Information 3.

## Data Availability

Data are available through the institutional medical charts database with relevant approvals. The datasets used and/or analyzed during the current study are available from the corresponding author on reasonable request.

## Abbreviations

PHH: Palmar hyperhidrosis
RFA: Radiofrequency ablation
VATS: Video-assisted thoracoscopic sympathectomy
CI: Confidence interval
OR: Odds ratio
HR: Hazard ratio
HDSS: Hyperhidrosis Disease Severity Scale
QOL: Quality of life questionnaire
SD: Standard deviation
SMD: Standardized mean difference
