# Supplementary material for: Efficacy and safety of radiofrequency ablation versus surgical sympathectomy in palmar hyperhidrosis
Source: Sci Rep. 2024 Apr 1;14:7620. doi: 10.1038/s41598-024-57834-0 (PMC10982298; doi:10.1038/s41598-024-57834-0)
Supplement: Supplementary file 1 — Supplementary Information 1. [file 41598_2024_57834_MOESM1_ESM.docx]

# Supplemental Appendix

**Table S1:** Baseline characteristics and Outcome variable in two cohort.

|  | **All patients (n=807)** | **RFA (n=351)** | **VATS (n=456)** | **SMD** | $\boldsymbol{x}^{\boldsymbol{2}}$**/t** | **Odds Ratio (95% CI)** | **P-Value** |
| --- | --- | --- | --- | --- | --- | --- | --- |
| **Baseline characteristics** |  |  |  |  |  |  |  |
| Age, mean (SD), year | 23.92 (6.22) | 25.13 (6.69) | 22.99 (5.65) | 0.345 | -4.805 | 0.445 (0.3010-1.264) | < 0.001 |
| Female, n (%) | 402 (49.8) | 187 (53.3) | 215 (47.1) | 0.123 | 2.978 | 1.130 (0.984-1.297) | 0.089 |
| Family history, n (%) | 336 (41.6) | 141 (40.2) | 195 (42.8) | 0.053 | 0.548 | 0.939 (0.796-1.109) | 0.472 |
| HDSS in Preoperative, n (%) * |  |  |  | 0.191 | 6.207 | 1.147 (0.906-1.452) | 0.256 |
| 1 | 0 (0) | 0 (0.0) | 0 (0.0) |  |  |  |  |
| 2 | 42 (5.2) | 26 (7.4) | 16 (3.5) |  |  |  |  |
| 3 | 384 (47.6) | 161 (45.9) | 223 (48.9) |  |  |  |  |
| 4 | 381 (47.2) | 164 (46.7) | 217 (47.6) |  |  |  |  |
| QOL in Preoperative, n (%) |  |  |  | 0.113 | 2.533 | 1.188 (0.958-1.475) | 0.117 |
| 20-35 | 0 (0) | 0 (0.0) | 0 (0.0) |  |  |  |  |
| 36-51 | 0 (0) | 0 (0.0) | 0 (0.0) |  |  |  |  |
| 52-68 | 79 (9.8) | 38 (10.8) | 41 (9.0) |  |  |  |  |
| 69-84 | 384 (47.6) | 174 (49.6) | 210 (46.1) |  |  |  |  |
| 85-100 | 344 (42.6) | 139 (39.6) | 205 (45.0) |  |  |  |  |
| **Outcome variable** |  |  |  |  |  |  |  |
| Efficacy, n (%) | 697 (86.4) | 282 (80.3) | 415 (91.0) | 0.308 | 19.17 | 0.404 (0.267-0.611) | < 0.001 |
| HDSS in Postoperative, n (%) * |  |  |  | 0.270 | 14.757 | 0.745 (0.612-0.907) | 0.003 |
| 1 | 686 (85.0) | 280 (79.8) | 406 (89.0) |  |  |  |  |
| 2 | 52 (6.4) | 31 (8.8) | 21 (4.6) |  |  |  |  |
| 3 | 39 (4.8) | 25 (7.1) | 14 (3.1) |  |  |  |  |
| 4 | 30 (3.7) | 15 (4.3) | 15 (3.3) |  |  |  |  |
| QOL in Postoperative, n (%) |  |  |  | 0.297 | 18.047 | 0.676 (0.556-0.823) | < 0.001 |
| 20-35 | 615 (76.2) | 248 (70.7) | 367 (80.5) |  |  |  |  |
| 36-51 | 114 (14.1) | 53 (15.1) | 61 (13.4) |  |  |  |  |
| 52-68 | 62 (7.7) | 38 (10.8) | 24 (5.3) |  |  |  |  |
| 69-84 | 11 (1.4) | 9 (2.6) | 2 (0.4) |  |  |  |  |
| 85-100 | 5 (0.6) | 3 (0.9) | 2 (0.4) |  |  |  |  |
| Symptom recurrence, n (%) | 101 (12.5) | 64 (18.2) | 37 (8.1) | 0.303 | 18.55 | 2.247 (1.537-3.286) | < 0.001 |
| Palm dry in postoperative, n (%) |  | 43 (12.3) | 84 (18.4) | 0.172 | 5.694 | 0.665 (0.473-0.934) | 0.019 |
| Compensatory hyperhidrosis, n (%) | 523 (64.8) | 235 (67.0) | 288 (63.2) | 0.080 | 1.252 | 1.060 (0.958-1.173) | 0.266 |
| Patient satisfaction, n (%) |  |  |  | 0.127 | 3.242 | 1.055 (0.890-1.251) | 0.539 |
| Dissatisfaction | 29 (3.6) | 16 (4.6) | 13 (2.9) |  |  |  |  |
| Moderate | 90 (11.2) | 42 (12.0) | 48 (10.5) |  |  |  |  |
| Satisfaction | 264 (32.7) | 106 (30.2) | 158 (34.6) |  |  |  |  |
| Complete satisfaction | 424 (29.4) | 187 (53.3) | 237 (52.0) |  |  |  |  |
| Complication, n (%) |  |  |  | 0.421 | 32.789 | 1.204 (1.071-1.353) | 0.001 |
| No Complication | 757 (93.8) | 338 (96.3) | 419 (91.9) |  |  |  |  |
| Dyspnea | 1 (0.1) | 1 (0.3) | 0 (0.0) |  |  |  |  |
| Axillary pain | 1 (0.1) | 1 (0.3) | 0 (0.0) |  |  |  |  |
| Acute Chest Syndrome | 3 (0.4) | 3 (0.9) | 0 (0.0) |  |  |  |  |
| Pneumothorax | 19 (2.4) | 3 (0.9) | 16 (3.5) |  |  |  |  |
| Bradycardia | 1 (0.1) | 1 (0.3) | 0 (0.0) |  |  |  |  |
| Waist discomfort | 3 (0.4) | 3 (0.9) | 0 (0.0) |  |  |  |  |
| Incisional pain | 8 (1.0) | 0 (0.0) | 8 (1.8) |  |  |  |  |
| Incisional paralysis | 1 (0.1) | 0 (0.0) | 1 (0.2) |  |  |  |  |
| Shoulder-back pain | 3 (0.4) | 1 (0.3) | 2 (0.4) |  |  |  |  |
| Subcutaneous Emphysema | 1 (0.1) | 0 (0.0) | 1 (0.2) |  |  |  |  |
| Pleural effusion | 9 (1.1) | 0 (0.0) | 9 (2.0) |  |  |  |  |
| Pain after discharge, (%) | 166 (20.6) | 32 (9.1) | 134 (29.4) | 0.532 | 49.871 | 0.310 (0.217-0.445) | <0.001 |
| Palm temperature rise, n (%) | 473 (58.6) | 253 (72.1) | 220 (48.2) | 0.502 | 46.445 | 1.494 (1.331-1.676) | <0.001 |
| Length of stay, mean (SD) | 2.90 (1.58) | 2.43 (1.22) | 3.26 (1.72) | 0.557 | 8.015 | 0.108 (0.617-1.032) | <0.001 |
| Hospital costs, mean (SD) | 1739.6 (4949.7) | 1013.9 (376.6) | 2298.1 (6524.7) | 0.278 | 4.194 | 306.2 (682.4-1885.9) | <0.001 |
| * 1: My sweating is never noticeable and does not interfere with my daily activities; 2: My sweating is tolerable but sometimes it interferes with my daily activities; 3: My sweating is barely tolerable and frequently interferes with my daily activities; 4: My sweating is intolerable and always interferes with my daily activities.  VATS: video-assisted thoracoscopic sympathectomy; RFA: radiofrequency ablation; HDSS: Hyperhidrosis Disease Severity Scale; QOL: quality of life questionnaire. | | | | | | | |

**Table S2**. Comparison of radiofrequency ablation (RFA) versus video-assisted thoracoscopic sympathectomy (VATS) for primary hyperhidrosis according to the HDSS and QOL in preoperative and postoperative with matched cohort.

|  | RFA (n=312) | | | VATS (n=312) | | |
| --- | --- | --- | --- | --- | --- | --- |
|  | Preoperative | Postoperative | P-value | Preoperative | Postoperative | P-value |
| HDSS* |  |  |  |  |  |  |
| 1 | 0 (0.0) | 245 (78.5) | <0.001 | 0 (0.0) | 275 (88.1) | <0.001 |
| 2 | 19 (6.1) | 30 (9.6) | 0.102 | 14 (4.5) | 16 (5.1) | 0.708 |
| 3 | 144 (46.2) | 25 (8.0) | <0.001 | 143 (45.8) | 9 (2.9) | <0.001 |
| 4 | 149 (47.8) | 12 (3.8) | <0.001 | 155 (49.7) | 12 (3.8) | <0.001 |
| QOL |  |  |  |  |  |  |
| 20-35 | 0 (0.0) | 217 (69.6) | <0.001 | 0 (0.0) | 251 (80.4) | <0.001 |
| 36-51 | 0 (0.0) | 50 (16.0) | <0.001 | 0 (0.0) | 42 (13.5) | <0.001 |
| 52-68 | 34 (10.9) | 35 (11.2) | 0.898 | 33 (10.6) | 17 (5.4) | 0.018 |
| 69-84 | 152 (48.7) | 7 (2.2) | <0.001 | 153 (49.0) | 2 (0.6) | <0.001 |
| 85-100 | 126 (40.4) | 3 (1.0) | <0.001 | 126 (40.4) | 0 (0.0) | <0.001 |
| * 1: My sweating is never noticeable and does not interfere with my daily activities; 2: My sweating is tolerable but sometimes it interferes with my daily activities; 3: My sweating is barely tolerable and frequently interferes with my daily activities; 4: My sweating is intolerable and always interferes with my daily activities.  VATS: video-assisted thoracoscopic sympathectomy; RFA: radiofrequency ablation; HDSS: Hyperhidrosis Disease Severity Scale; QOL: quality of life questionnaire. | | | | | | |
