# Supplementary material for: Efficacy and safety of radiofrequency ablation versus surgical sympathectomy in palmar hyperhidrosis
Source: Sci Rep. 2024 Apr 1;14:7620. doi: 10.1038/s41598-024-57834-0 (PMC10982298; doi:10.1038/s41598-024-57834-0)
Supplement: Supplementary file 2 — Supplementary Information 2. [file 41598_2024_57834_MOESM2_ESM.docx]

# Statistical Analysis Plan

**Study Title:** Efficacy and safety of radiofrequency ablation versus video-assisted thoracoscopic sympathectomy in palmar hyperhidrosis: a multicenter cohort study

**Statistical Analysis Plan Authors:** Yiyue Zhong and Jing Tang

**Statistical Analysis Plan Date:** 10/13/2020

The objective of this analysis plan is to provide guide to our analyst when conducting the study. Most of the content will be included in the manuscript in order to guide researchers who want to replicate our findings or conduct similar studies. We also provided justifications for our methods and decisions, which the code of statistical analysis using R software, so other researchers can make a choice or adjust their methods accordingly.

**TABLE OF CONTENTS**

[Statistical Analysis Plan 1](#_Toc98061261)

[1. BACKGROUND AND OBJECTIVES 5](#_Toc98061262)

[2. STUDY DESIGN AND DATA SOURCE 8](#_Toc98061263)

[3. STUDY POPULATION 9](#_Toc98061264)

[4. MEASUREMENTS 10](#_Toc98061265)

[4.1 Baseline Characteristics 10](#_Toc98061266)

[4.2 Follow up and Outcomes 12](#_Toc98061267)

[4.3 Missing Data 13](#_Toc98061268)

[5. STATISTICAL METHODS 14](#_Toc98061269)

[5.1 Statistical Analyses 14](#_Toc98061270)

[5.2 Subgroup Analyses 14](#_Toc98061271)

[6. LIMITATIONS 16](#_Toc98061272)

[References 17](#_Toc98061273)

**Key Definition**
Index Date (variable name index Time) the date patients with palmar hyperhidrosis received surgery treatment.

**Baseline Period**
Any time before and including the index date used to establish a patient’s medical history.

**Study Period**
The study period will be March 1, 2015, and December 31, 2019. The last day of follow up is December 31, 2019. Because we will require patients to have at least 1 year of follow up, the last day of index procedure will be December 31, 2020.

1. **BACKGROUND AND OBJECTIVES**

Hyperhidrosis refers to sweating exceeding physiological needs, and is considered to be a disease of the autonomic nervous system, with the specific pathogenesis unclear.^1-3^ A recent study shows that the cholinergic receptor nicotinic alpha 1 subunit and activin a receptor type 1 maybe involvement of the pathogenesis of primary hyperhidrosis.^4,5^ The main involved symptom parts include hand, axilla, craniofacial region, and feet.^6,7^ Hyperhidrosis causes great troubles to patients' social life and work, and even depression in severe cases; severely affected patients have skin maceration and secondary microbial infections.^8-11^ Hyperhidrosis condition can be primary or secondary.^12^ Primary hyperhidrosis is excessive and uncontrollable sweating without any discernible cause.^8^ Secondary hyperhidrosis can be caused by some diseases or drugs, which can affect all parts of the body.^9,13^ A previous study reported that a prevalence of 2.8% in the United States, which would correspond to 7.8 million American individuals with hyperhidrosis.^14^ A nationwide survey has been performed, a prevalence of 2.08% in China, and positive family history was found in 25.4% of hyperhidrosis cases.^15^ More than half of hyperhidrosis symptom parts in the palms,^14^ even more than that.^15^

For hyperhidrosis of treatment, the options are divided into surgical and non-surgical options.^16,17^ Non-surgical treatment includes injections of botulinum toxin,^8^ topical antiperspirants (aluminum chloride hexahydrate),^18,19^ laser treatment,^20^ and oral medications (anticholinergics, beta-blockers, and benzodiazepines),^17^ and all these treatments have their limitations and higher the rate of recurrence. Therefore, surgery may be considered as a last resort when more conservative treatments have failed.^17^ Local surgical techniques there is excision, curettage, liposuction, or a combination of these techniques, and video-assisted thoracoscopic sympathectomy (VATS). However, more compensatory hyperhidrosis occurs after the VATS,^17^ and it requires general anesthesia and has more tremendous trauma than other options.^11,21-23^

As a minimally invasive therapies,^23^ radiofrequency ablation (RFA) has many satisfactory advantages such as more minor trauma and quick recovery.^24,25^ The therapeutic mechanism of RFA is a thermal effect, for tissue coagulation without causing neuromuscular excitation or pain.^26^ At present, RFA has been widely used to treat tumors, chronic pain, and has achieved remarkable results.^27^ A recent review and meta‑analysis show that the RFA is effective for hyperhidrosis treatment.^17^ However, only two studies were compared between RFA and VATS.^16,28^ In 2013, The first clinical study to evaluate the role of RFA and compare it with the VATS for palmar hyperhidrosis (PHH) reported that RFA has long-term patient satisfaction, which a success rate of 75% for treating hyperhidrosis in 46 patients.^16^ Another non-randomized controlled clinical trial supports the view of surgical sympathectomy as the gold-standard treatment in severe cases of PHH in a small series of cases.^28^ These studies with limited clinical data have not provided strong evidence for the management of hyperhidrosis.^18^ However, it is challenging to design a randomized controlled study to assess the effect and safety between the RFA and VATS in patients with PHH due to making randomization quite complex.^17^

We conducted a large multicenter cohort study based nationwide to compare the efficacy and safety between RFA and VATS in patients with PHH, to improve clinical practice.

**ABBREVIATIONS**

PHH: Palmar Hyperhidrosis

RFA: Radiofrequency Ablation

VATS: Video-assisted Thoracoscopic Sympathectomy

CI: Confidence interval

OR: odds ratios

HR: Hazard Ratio, a measure of relative risk used to compare different treatments; calculated from time-to-event Cox proportional hazards regression

HDSS: Hyperhidrosis Disease Severity Scale

QOL: quality of life questionnaire

SD: standard deviation

SMD: standardized mean differences

1. **STUDY DESIGN AND DATA SOURCE**

The nationwide of China comparative efficacy research to assess the use of RFA and VATS for PHH was an investigator-initiated, controlled cohort study done at 14 centers in China between March 4, 2015, and December 31, 2019.

1. **STUDY POPULATION**

Eligible participants were aged 14 years or older, were diagnosed as primary PHH who are undergoing treatment options with RFA or VATS. Exclusions included patients who are receiving medical treatment, non-palmar hyperhidrosis, secondary hyperhidrosis, and non-interventional treatment for hyperhidrosis.^16^

1. **MEASUREMENTS**
   1. **Baseline Characteristics**

The treatment options were done according to a prespecified analysis plan within between RFA and VATS framework. Per a prospective exposure factor definition, patients excluded who were secondary hyperhidrosis before RFA or VATS.

Baseline characteristics include demographic data, clinical disease (Hyperhidrosis Disease Severity Scale [HDSS]),^1^ quality of life (QOL) questionnaire,^28^ and family history were collected at preoperative from the medical charts each of participation center. The HDSS questionnaire are consists of four statements, each receiving a score of 1 to 4, with 1 being the mildest grade and 4 the worst.^29^ The QOL questionnaire is consists of twenty statements, each receiving a score of 1 to 5, with 20 being the mildest grade and 100 the worst. Outcomes variable included the clinical efficacy,^16^ safety (intraoperative and postoperative complication [e.g., dyspnea, pneumothorax, incisional paralysis or pain, acute chest syndrome, subcutaneous emphysema, pleural effusion, axillary pain, waist unwell, shoulder-back pain, bradycardia, nasal obstruction, nerve injury, and intercostal neuralgia]),^30,31^ patient satisfaction (chief complaint from patients), HDSS and QOL questionnaire,^32^ compensatory hyperhidrosis,^32^ and symptom recurrence ^6^ follow-up data of postoperative months 1, 3, 6, and 12 (1 year), as previously reported.^16,32^ For patient satisfaction is used the chief complaint of patients by phone call and return follow-up (dissatisfaction, moderate, satisfaction, complete satisfaction) to respectively evaluated. To evaluate the postoperative QOL is used the last data of follow-up questionnaire. If the endpoint review period was longer than one year at the follow-up, we used one year after treatment as the endpoint indicator. All follow-up was implemented by an external blinded endpoint committee (setting at the principal investigator institution) after ethics approval.

- 1. **Follow up and Outcomes**

The primary outcome was the clinical efficacy in 1-year. The success of clinical efficacy was defined as complete remission after treatment for PHH, and the ineffective treatment was defined as no remission or a few partial remissions after treatment for PHH. Surgical failure was defined as nothing improvement for hyperhidrosis symptoms in postoperative. The HDSS and QOL were assessed respectively in preoperative and postoperative as a component of the primary outcome were determined. The secondary outcomes were including symptom recurrence, complication, compensatory hyperhidrosis, patient satisfaction, length of stay, and hospital costs.

- 1. **Missing Data**

The patients with the loss to follow-up will be excluded from the study analysis.

1. **STATISTICAL METHODS**
   1. **Statistical Analyses**

Statistical analyses were carried out using R 3.6.3 software.

The RFA and VATS cohorts were analyzed separately for HDSS and QOL in preoperative and postoperative. The patients with the loss to follow-up will be excluded from the study analysis. In patients with PHH, baseline characteristics were compared between patients receiving RFA and VATS using standardized mean differences (SMD). The propensity score for receiving RFA was estimated using a logistic regression model. Covariates included in the model were demographics (age and sex), family history, HDSS and QOL in preoperative. Propensity-score matching was implemented using a nearest-neighbor strategy, with a minimum caliper of 0.1.^33,34^ The caliper for the matching was specified in the nearest-neighbor strategy if the unspecified approach did not result in satisfactory balance.^35^ The ratio was 1 patient receiving RFA matched with 1 patients receiving VATS. The SMD was used to assess the balance of baseline covariates between the RFA and VATS groups in the matched cohort. An SMD of less than 0.10 indicated a good balance.^36^ In the matched cohort, the non-normally distributed of the length of stay and hospitalization costs were converted to categorical variables based on the median. The primary outcome and secondary outcomes for compared the distributions of categorical variables using the chi-square test in the unmatched cohort and a logistic-regression models in the matched cohort, which is reported with odds ratios (OR) and 95% confidence intervals (CI) for two treatment group.

- 1. **Subgroup Analyses**

To test whether the findings of the patient-level analysis might be due to a causal effect of RFA, we used a further adjusted time-varying Cox proportional hazard model to estimate the hazard ratio for ineffective treatment in the matched cohort.

1. **LIMITATIONS**

This study has several limitations. First, an observational study to evaluating the clinical efficacy of RFA and VATS are potentially subjected to selection bias, and randomized studies are the gold standard for assessment of clinical efficacy.^37^ Even though the balance was achieved in each cohort by propensity matching score, but it is still possible that patients selected for RFA differed in terms of treatment history compared with patients receiving VATS, and seven centers did not perform RFA for PHH. However, sympathectomy is the last resort of treatment when conservative treatments are a failure or intolerable,^18^ which treatment history did not affect the clinical outcome of sympathectomy.^3,4^ Second, the proposed different follow-up times were observed for RFA or VATS treatment effect with HDSS and QOL needs to be independently assessed.^32^ In this context, it is essential to note that the endpoint assessment for treatment of clinical efficacy for long-term outcomes might need more concerned.^17^ Third, this study lack data were indicating ethnicity. Although ethnicity could potentially affect the study results, the study population only is yellow. Finally, missing data possibly influence results, we are not sure whether the inclusion of lost to follow-up data will affect the final analysis results in this study.

# References

1. Gabes M, Jourdan C, Schramm K, et al. Hyperhidrosis Quality of Life Index (HidroQoL©): further validation and clinical application in patients with axillary hyperhidrosis using data from a phase III randomized controlled trial. *The British journal of dermatology.* 2020.

2. Wade R, Llewellyn A, Jones-Diette J, et al. Interventional management of hyperhidrosis in secondary care: a systematic review. *The British journal of dermatology.* 2018;179(3):599-608.

3. Schote AB, Schiel F, Schmitt B, et al. Genome-wide linkage analysis of families with primary hyperhidrosis. *PLoS One.* 2020;15(12):e0244565.

4. Lin JB, Kang MQ, Huang LP, Zhuo Y, Li X, Lai FC. CHRNA1 promotes the pathogenesis of primary focal hyperhidrosis. *Molecular and cellular neurosciences.* 2021:103598.

5. Lin JB, Chen JF, Lai FC, et al. Involvement of activin a receptor type 1 (ACVR1) in the pathogenesis of primary focal hyperhidrosis. *Biochem Biophys Res Commun.* 2020;528(2):299-304.

6. Purtuloğlu T, Deniz S, Atım A, Tekindur Ş, Gürkök S, Kurt E. A new target of percutaneus sympathic radiofrequency thermocoagulation for treatment of palmar hyperhidrosis: T4. *Agri : Agri (Algoloji) Dernegi'nin Yayin organidir = The journal of the Turkish Society of Algology.* 2013;25(1):36-40.

7. Lecouflet M, Leux C, Fenot M, Célerier P, Maillard H. Duration of efficacy increases with the repetition of botulinum toxin A injections in primary palmar hyperhidrosis: a study of 28 patients. *J Am Acad Dermatol.* 2014;70(6):1083-1087.

8. Heckmann M, Ceballos-Baumann AO, Plewig G. Botulinum toxin A for axillary hyperhidrosis (excessive sweating). *N Engl J Med.* 2001;344(7):488-493.

9. Walling HW. Primary hyperhidrosis increases the risk of cutaneous infection: a case-control study of 387 patients. *J Am Acad Dermatol.* 2009;61(2):242-246.

10. Hamm H, Naumann MK, Kowalski JW, Kütt S, Kozma C, Teale C. Primary focal hyperhidrosis: disease characteristics and functional impairment. *Dermatology.* 2006;212(4):343-353.

11. Nyamekye IK. Current therapeutic options for treating primary hyperhidrosis. *European journal of vascular and endovascular surgery : the official journal of the European Society for Vascular Surgery.* 2004;27(6):571-576.

12. McConaghy JR, Fosselman D. Hyperhidrosis: Management Options. *American family physician.* 2018;97(11):729-734.

13. Galadari H, Galadari I, Smit R, Prygova I, Redaelli A. Treatment approaches and outcomes associated with the use of abobotulinumtoxinA for the treatment of hyperhidrosis: a systematic review. *J Am Acad Dermatol.* 2020.

14. Strutton DR, Kowalski JW, Glaser DA, Stang PE. US prevalence of hyperhidrosis and impact on individuals with axillary hyperhidrosis: results from a national survey. *J Am Acad Dermatol.* 2004;51(2):241-248.

15. Lai FC, Tu YR, Li YP, et al. Nation wide epidemiological survey of primary palmar hyperhidrosis in the People's Republic of China. *Clinical autonomic research : official journal of the Clinical Autonomic Research Society.* 2015;25(2):105-108.

16. Purtuloglu T, Atim A, Deniz S, et al. Effect of radiofrequency ablation and comparison with surgical sympathectomy in palmar hyperhidrosis. *European journal of cardio-thoracic surgery : official journal of the European Association for Cardio-thoracic Surgery.* 2013;43(6):e151-154.

17. Hasimoto FN, Cataneo DC, Hasimoto EN, Ximenes AMG, Cataneo AJM. Radiofrequency in the treatment of primary hyperhidrosis: systematic review and meta-analysis. *Clinical autonomic research : official journal of the Clinical Autonomic Research Society.* 2020;30(2):111-120.

18. Nawrocki S, Cha J. The etiology, diagnosis, and management of hyperhidrosis: A comprehensive review: Therapeutic options. *J Am Acad Dermatol.* 2019;81(3):669-680.

19. Gee S, Yamauchi PS. Nonsurgical management of hyperhidrosis. *Thoracic surgery clinics.* 2008;18(2):141-155.

20. Cervantes J, Perper M, Eber AE, Fertig RM, Tsatalis JP, Nouri K. Laser treatment of primary axillary hyperhidrosis: a review of the literature. *Lasers in medical science.* 2018;33(3):675-681.

21. Atkinson JL, Fode-Thomas NC, Fealey RD, Eisenach JH, Goerss SJ. Endoscopic transthoracic limited sympathotomy for palmar-plantar hyperhidrosis: outcomes and complications during a 10-year period. *Mayo Clin Proc.* 2011;86(8):721-729.

22. Cruddas L, Baker DM. Treatment of primary hyperhidrosis with oral anticholinergic medications: a systematic review. *Journal of the European Academy of Dermatology and Venereology : JEADV.* 2017;31(6):952-963.

23. Alric P, Branchereau P, Berthet JP, Léger P, Mary H, Mary-Ané C. Video-assisted thoracoscopic sympathectomy for palmar hyperhidrosis: results in 102 cases. *Ann Vasc Surg.* 2002;16(6):708-713.

24. Abd-Elsayed A, Nguyen S, Fiala K. Radiofrequency Ablation for Treating Headache. *Current pain and headache reports.* 2019;23(3):18.

25. Guo L, Kubat NJ, Nelson TR, Isenberg RA. Meta-analysis of clinical efficacy of pulsed radio frequency energy treatment. *Ann Surg.* 2012;255(3):457-467.

26. Starr JB, Gold L, McCormick Z, Suri P, Friedly J. Trends in lumbar radiofrequency ablation utilization from 2007 to 2016. *The spine journal : official journal of the North American Spine Society.* 2019;19(6):1019-1028.

27. Friedman M, Mikityansky I, Kam A, et al. Radiofrequency ablation of cancer. *Cardiovasc Intervent Radiol.* 2004;27(5):427-434.

28. Garcia Franco CE, Perez-Cajaraville J, Guillen-Grima F, España A. Prospective study of percutaneous radiofrequency sympathicolysis in severe hyperhidrosis and facial blushing: efficacy and safety findings. *European journal of cardio-thoracic surgery : official journal of the European Association for Cardio-thoracic Surgery.* 2011;40(4):e146-151.

29. Solish N, Bertucci V, Dansereau A, et al. A comprehensive approach to the recognition, diagnosis, and severity-based treatment of focal hyperhidrosis: recommendations of the Canadian Hyperhidrosis Advisory Committee. *Dermatologic surgery : official publication for American Society for Dermatologic Surgery [et al].* 2007;33(8):908-923.

30. Lai YT, Yang LH, Chio CC, Chen HH. Complications in patients with palmar hyperhidrosis treated with transthoracic endoscopic sympathectomy. *Neurosurgery.* 1997;41(1):110-113; discussion 113-115.

31. Puffer RC, Bishop AT, Spinner RJ, Shin AY. Bilateral brachial plexus injury after MiraDry® procedure for axillary hyperhidrosis: a case report. *World neurosurgery.* 2019.

32. Romero FR, Cataneo DC, Cataneo AJM. Outcome of Percutaneous Radiofrequency Thoracic Sympathectomy for Palmar Hyperhidrosis. *Seminars in thoracic and cardiovascular surgery.* 2018;30(3):362-366.

33. Austin PC. Optimal caliper widths for propensity-score matching when estimating differences in means and differences in proportions in observational studies. *Pharmaceutical statistics.* 2011;10(2):150-161.

34. Ouldali N, Toubiana J, Antona D, et al. Association of Intravenous Immunoglobulins Plus Methylprednisolone vs Immunoglobulins Alone With Course of Fever in Multisystem Inflammatory Syndrome in Children. *Jama.* 2021.

35. Reynolds HR, Adhikari S, Pulgarin C, et al. Renin-Angiotensin-Aldosterone System Inhibitors and Risk of Covid-19. *N Engl J Med.* 2020;382(25):2441-2448.

36. Austin PC, Stuart EA. Moving towards best practice when using inverse probability of treatment weighting (IPTW) using the propensity score to estimate causal treatment effects in observational studies. *Statistics in medicine.* 2015;34(28):3661-3679.

37. Agoritsas T, Merglen A, Shah ND, O'Donnell M, Guyatt GH. Adjusted Analyses in Studies Addressing Therapy and Harm: Users' Guides to the Medical Literature. *Jama.* 2017;317(7):748-759.
