# Supplementary material for: Efficacy and safety of radiofrequency ablation versus surgical sympathectomy in palmar hyperhidrosis
Source: Sci Rep. 2024 Apr 1;14:7620. doi: 10.1038/s41598-024-57834-0 (PMC10982298; doi:10.1038/s41598-024-57834-0)
Supplement: Supplementary file 3 — Supplementary Information 3. [file 41598_2024_57834_MOESM3_ESM.docx]

The code of statistical analysis using R software

> library(MatchIt)

> library(table1)

> library(epiDisplay)

> library(tableone)

> library(survey)

> library(reshape2)

> library(ggplot2)

> library("foreign", lib.loc="C:/Program Files/R/R-3.6.3/library")

> mydata <- read.spss ("C:/Users/DELL/Desktop/R Mydata/Matched Cohort.sav", to.data.frame = TRUE)

> mydata$Group = factor(mydata$Group)

> mydata$Outcome = factor(mydata$Outcome)

> mydata$Recurrence = factor(mydata$Recurrence)

> mydata$Time = as.numeric(mydata$Time)

> mydata$V1 = as.numeric(mydata$V1)

> mydata$V2 = factor(mydata$V2)

> mydata$V3 = factor(mydata$V3)

> mydata$V4 = as.numeric(mydata$V4)

> mydata$V4_1 = factor(mydata$V4_1)

> mydata$V5 = factor(mydata$V5)

> mydata$V5_1 = factor(mydata$V5_1)

> mydata$V5_2 = factor(mydata$V5_2)

> mydata$V5_3 = factor(mydata$V5_3)

> mydata$V5_4 = factor(mydata$V5_4)

> mydata$V6 = factor(mydata$V6)

> mydata$V6_1 = factor(mydata$V6_1)

> mydata$V6_2 = factor(mydata$V6_2)

> mydata$V6_3 = factor(mydata$V6_3)

> mydata$V6_4 = factor(mydata$V6_4)

> mydata$V7 = factor(mydata$V7)

> mydata$V7_1 = factor(mydata$V7_1)

> mydata$V7_2 = factor(mydata$V7_2)

> mydata$V7_3 = factor(mydata$V7_3)

> mydata$V7_4 = factor(mydata$V7_4)

> mydata$V7_5 = factor(mydata$V7_5)

> mydata$V8 = factor(mydata$V8)

> mydata$V8_1 = factor(mydata$V8_1)

> mydata$V8_2 = factor(mydata$V8_2)

> mydata$V8_3 = factor(mydata$V8_3)

> mydata$V8_4 = factor(mydata$V8_4)

> mydata$V8_5 = factor(mydata$V8_5)

> mydata$V9 = factor(mydata$V9)

> mydata$V10 = factor(mydata$V10)

> mydata$V10_1 = factor(mydata$V10_1)

> mydata$V10_2 = factor(mydata$V10_2)

> mydata$V10_3 = factor(mydata$V10_3)

> mydata$V11 = factor(mydata$V11)

> mydata$V11_1 = factor(mydata$V11_1)

> mydata$V11_2 = factor(mydata$V11_2)

> mydata$V11_3 = factor(mydata$V11_3)

> mydata$V11_4 = factor(mydata$V11_4)

> mydata$V11_5 = factor(mydata$V11_5)

> mydata$V12 = factor(mydata$V12)

> mydata$V12_1 = factor(mydata$V12_1)

> mydata$V12_0 = factor(mydata$V12_0)

> mydata$V12_2 = factor(mydata$V12_2)

> mydata$V12_3 = factor(mydata$V12_3)

> mydata$V13 = factor(mydata$V13)

> mydata$V13_1 = factor(mydata$V13_1)

> mydata$V13_2 = factor(mydata$V13_2)

> mydata$V13_3 = factor(mydata$V13_3)

> mydata$V13_4 = factor(mydata$V13_4)

> mydata$V13_5 = factor(mydata$V13_5)

> mydata$V13_6 = factor(mydata$V13_6)

> mydata$V13_7 = factor(mydata$V13_7)

> mydata$V13_8 = factor(mydata$V13_8)

> mydata$V13_9 = factor(mydata$V13_9)

> mydata$V13_10 = factor(mydata$V13_10)

> mydata$V13_11 = factor(mydata$V13_11)

> mydata$V13_0 = factor(mydata$V13_0)

> mydata$V14 = factor(mydata$V14)

> mydata$V15 = factor(mydata$V15)

> mydata$costs = as.numeric(mydata$costs)

> mydata$V_costs = factor(mydata$V_costs)

> mydata$Centre = factor(mydata$Centre)

> m.out <- matchit(Group ~ V1 + V2 + V3 + V5 + V7,data = mydata, method = "nearest",ratio =1,caliper=0.1)

> summary (m.out)

> plot (m.out, type = "jitter")

> plot (m.out, type = "hist")

> matchdata=match.data(m.out)

> vars <- c("V1","V2","V3","V5","V7")

> tabUnmatched <- CreateTableOne(vars = vars, strata = "Group", data = mydata, test = FALSE)

> print(tabUnmatched, smd = TRUE)

> tabUnmatched <- CreateTableOne(vars = vars, strata = "Group", data = matchdata, test = FALSE)

> print(tabUnmatched, smd = TRUE)

> vars <- c("V1", "V2", "V3", "V5", "V7", "Efficacy", "Recurrence", "V4", "V4_1", "V6", "V8", "V9", "V11", "V12", "V13", "V13_0", "V14", "V15", "costs", "V_costs")

> tabUnmatched <- CreateTableOne(vars = vars, strata = "Group", data = mydata, test = FALSE)

> print(tabUnmatched, smd = TRUE)

tabmatched <- CreateTableOne(vars = vars, strata = "Group", data = matchdata, test = FALSE)

> print(tabmatched, smd = TRUE)

> library(epiDisplay)

> mytable <- table(matchdata$Group,matchdata$V2)

> tabpct(matchdata$Group,matchdata$V2)

> tabpct(matchdata$Group,matchdata$V3)

> tabpct(matchdata$Group,matchdata$V5)

> tabpct(matchdata$Group,matchdata$V7)

> summ(mydata)

> tabpct(matchdata$Group,matchdata$V10)

> tabpct(matchdata$Group,matchdata$Efficacy)

> cc(matchdata$Group,matchdata$Efficacy)

> tabpct(matchdata$Group,matchdata$V6)

> cc(matchdata$Group,matchdata$V6_1)

> cc(matchdata$Group,matchdata$V6_2)

> cc(matchdata$Group,matchdata$V6_3)

> cc(matchdata$Group,matchdata$V6_4)

> tabpct(matchdata$Group,matchdata$V8)

> cc(matchdata$Group,matchdata$V8_1)

> cc(matchdata$Group,matchdata$V8_2)

> cc(matchdata$Group,matchdata$V8_3)

> cc(matchdata$Group,matchdata$V8_4)

> cc(matchdata$Group,matchdata$V8_5)

> tabpct(matchdata$Group,matchdata$Recurrence)

> cc(matchdata$Group,matchdata$Recurrence)

> tabpct(matchdata$Group,matchdata$V11)

> cc(matchdata$Group,matchdata$V11)

> tabpct(matchdata$Group,matchdata$V9)

> cc(matchdata$Group,matchdata$V9)

> tabpct(matchdata$Group,matchdata$V14)

> cc(matchdata$Group,matchdata$V14)

> tabpct(matchdata$Group,matchdata$V15)

> cc(matchdata$Group,matchdata$V15)

> tabpct(matchdata$Group,matchdata$V12)

> cc(matchdata$Group,matchdata$V12_0)

> cc(matchdata$Group,matchdata$V12_1)

> cc(matchdata$Group,matchdata$V12_2)

> cc(matchdata$Group,matchdata$V12_3)

> tabpct(matchdata$Group,matchdata$V13_0)

> cc(matchdata$Group,matchdata$V13_0)

> tabpct(matchdata$Group,matchdata$V4_1)

> cc(matchdata$Group,matchdata$V4_1)

> tabpct(matchdata$Group,matchdata$V_costs)

> cc(matchdata$Group,matchdata$V_costs)

> glmMatched <- glm(formula = (V10_1 == "yes") ~ Group + V1 + V2 + V3 + V5 + V7,

+ family = binomial(link = "logit"),

+ data = matchdata, control=list(maxit=100))

> glmUnmatched <- glm(formula = (V10_1 == "yes") ~ Group + V1 + V2 + V3 + V5 + V7,

+ family = binomial(link = "logit"),

+ data = mydata, control=list(maxit=100))

> resTogether <- list(Unmatched = ShowRegTable(glmUnmatched, printToggle = FALSE),

+ Matched = ShowRegTable(glmMatched, printToggle = FALSE))

> print(resTogether, quote = FALSE)

> glmMatched <- glm(formula = (V10_3 == "yes") ~ Group + V1 + V2 + V3 + V5 + V7,

+ family = binomial(link = "logit"),

+ data = matchdata, control=list(maxit=100))

> glmUnmatched <- glm(formula = (V10_3 == "yes") ~ Group + V1 + V2 + V3 + V5 + V7,

+ family = binomial(link = "logit"),

+ data = mydata, control=list(maxit=100))

> resTogether <- list(Unmatched = ShowRegTable(glmUnmatched, printToggle = FALSE),

+ Matched = ShowRegTable(glmMatched, printToggle = FALSE))

> print(resTogether, quote = FALSE)

> glmMatched <- glm(formula = (Efficacy == "no") ~ Group + V1 + V2 + V3 + V5 + V7,

+ family = binomial(link = "logit"),

+ data = matchdata, control=list(maxit=100))

> glmUnmatched <- glm(formula = (Efficacy == "no") ~ Group + V1 + V2 + V3 + V5 + V7,

+ family = binomial(link = "logit"),

+ data = mydata, control=list(maxit=100))

> resTogether <- list(Unmatched = ShowRegTable(glmUnmatched, printToggle = FALSE),

+ Matched = ShowRegTable(glmMatched, printToggle = FALSE))

> print(resTogether, quote = FALSE)

> glmMatched <- glm(formula = (Efficacy == "yes") ~ Group + V1 + V2 + V3 + V5 + V7,

+ family = binomial(link = "logit"),

+ data = matchdata, control=list(maxit=100))

> glmUnmatched <- glm(formula = (Efficacy == "yes") ~ Group + V1 + V2 + V3 + V5 + V7,

+ family = binomial(link = "logit"),

+ data = mydata, control=list(maxit=100))

> resTogether <- list(Unmatched = ShowRegTable(glmUnmatched, printToggle = FALSE),

+ Matched = ShowRegTable(glmMatched, printToggle = FALSE))

> print(resTogether, quote = FALSE)

> glmMatched <- glm(formula = (Recurrence == "yes") ~ Group + V1 + V2 + V3 + V5 + V7,

+ family = binomial(link = "logit"),

+ data = matchdata, control=list(maxit=100))

> glmUnmatched <- glm(formula = (Recurrence == "yes") ~ Group + V1 + V2 + V3 + V5 + V7,

+ family = binomial(link = "logit"),

+ data = mydata, control=list(maxit=100))

> resTogether <- list(Unmatched = ShowRegTable(glmUnmatched, printToggle = FALSE),

+ Matched = ShowRegTable(glmMatched, printToggle = FALSE))

> print(resTogether, quote = FALSE)

> glmMatched <- glm(formula = (V6_1 == "yes") ~ Group + V1 + V2 + V3 + V5 + V7,

+ family = binomial(link = "logit"),

+ data = matchdata, control=list(maxit=100))

> glmUnmatched <- glm(formula = (V6_1 == "yes") ~ Group + V1 + V2 + V3 + V5 + V7,

+ family = binomial(link = "logit"),

+ data = mydata, control=list(maxit=100))

> resTogether <- list(Unmatched = ShowRegTable(glmUnmatched, printToggle = FALSE),

+ Matched = ShowRegTable(glmMatched, printToggle = FALSE))

> print(resTogether, quote = FALSE)

> glmMatched <- glm(formula = (V6_2 == "yes") ~ Group + V1 + V2 + V3 + V5 + V7,

+ family = binomial(link = "logit"),

+ data = matchdata, control=list(maxit=100))

> glmUnmatched <- glm(formula = (V6_2 == "yes") ~ Group + V1 + V2 + V3 + V5 + V7,

+ family = binomial(link = "logit"),

+ data = mydata, control=list(maxit=100))

> resTogether <- list(Unmatched = ShowRegTable(glmUnmatched, printToggle = FALSE),

+ Matched = ShowRegTable(glmMatched, printToggle = FALSE))

> print(resTogether, quote = FALSE)

> glmMatched <- glm(formula = (V6_3 == "yes") ~ Group + V1 + V2 + V3 + V5 + V7,

+ family = binomial(link = "logit"),

+ data = matchdata, control=list(maxit=100))

> glmUnmatched <- glm(formula = (V6_3 == "yes") ~ Group + V1 + V2 + V3 + V5 + V7,

+ family = binomial(link = "logit"),

+ data = mydata, control=list(maxit=100))

> resTogether <- list(Unmatched = ShowRegTable(glmUnmatched, printToggle = FALSE),

+ Matched = ShowRegTable(glmMatched, printToggle = FALSE))

> print(resTogether, quote = FALSE)

> glmMatched <- glm(formula = (V6_4 == "yes") ~ Group + V1 + V2 + V3 + V5 + V7,

+ family = binomial(link = "logit"),

+ data = matchdata, control=list(maxit=100))

> glmUnmatched <- glm(formula = (V6_4 == "yes") ~ Group + V1 + V2 + V3 + V5 + V7,

+ family = binomial(link = "logit"),

+ data = mydata, control=list(maxit=100))

> resTogether <- list(Unmatched = ShowRegTable(glmUnmatched, printToggle = FALSE),

+ Matched = ShowRegTable(glmMatched, printToggle = FALSE))

> print(resTogether, quote = FALSE)

> glmMatched <- glm(formula = (V8_1 == "yes") ~ Group + V1 + V2 + V3 + V5 + V7,

+ family = binomial(link = "logit"),

+ data = matchdata, control=list(maxit=100))

> glmUnmatched <- glm(formula = (V8_1 == "yes") ~ Group + V1 + V2 + V3 + V5 + V7,

+ family = binomial(link = "logit"),

+ data = mydata, control=list(maxit=100))

> resTogether <- list(Unmatched = ShowRegTable(glmUnmatched, printToggle = FALSE),

+ Matched = ShowRegTable(glmMatched, printToggle = FALSE))

> print(resTogether, quote = FALSE)

> glmMatched <- glm(formula = (V8_2 == "yes") ~ Group + V1 + V2 + V3 + V5 + V7,

+ family = binomial(link = "logit"),

+ data = matchdata, control=list(maxit=100))

> glmUnmatched <- glm(formula = (V8_2 == "yes") ~ Group + V1 + V2 + V3 + V5 + V7,

+ family = binomial(link = "logit"),

+ data = mydata, control=list(maxit=100))

> resTogether <- list(Unmatched = ShowRegTable(glmUnmatched, printToggle = FALSE),

+ Matched = ShowRegTable(glmMatched, printToggle = FALSE))

> print(resTogether, quote = FALSE)

> glmMatched <- glm(formula = (V8_3 == "yes") ~ Group + V1 + V2 + V3 + V5 + V7,

+ family = binomial(link = "logit"),

+ data = matchdata, control=list(maxit=100))

> glmUnmatched <- glm(formula = (V8_3 == "yes") ~ Group + V1 + V2 + V3 + V5 + V7,

+ family = binomial(link = "logit"),

+ data = mydata, control=list(maxit=100))

> resTogether <- list(Unmatched = ShowRegTable(glmUnmatched, printToggle = FALSE),

+ Matched = ShowRegTable(glmMatched, printToggle = FALSE))

> print(resTogether, quote = FALSE)

> glmMatched <- glm(formula = (V8_4 == "yes") ~ Group + V1 + V2 + V3 + V5 + V7,

+ family = binomial(link = "logit"),

+ data = matchdata, control=list(maxit=100))

> glmUnmatched <- glm(formula = (V8_4 == "yes") ~ Group + V1 + V2 + V3 + V5 + V7,

+ family = binomial(link = "logit"),

+ data = mydata, control=list(maxit=100))

> resTogether <- list(Unmatched = ShowRegTable(glmUnmatched, printToggle = FALSE),

+ Matched = ShowRegTable(glmMatched, printToggle = FALSE))

> print(resTogether, quote = FALSE)

> glmMatched <- glm(formula = (V8_5 == "yes") ~ Group + V1 + V2 + V3 + V5 + V7,

+ family = binomial(link = "logit"),

+ data = matchdata, control=list(maxit=100))

> glmUnmatched <- glm(formula = (V8_5 == "yes") ~ Group + V1 + V2 + V3 + V5 + V7,

+ family = binomial(link = "logit"),

+ data = mydata, control=list(maxit=100))

> resTogether <- list(Unmatched = ShowRegTable(glmUnmatched, printToggle = FALSE),

+ Matched = ShowRegTable(glmMatched, printToggle = FALSE))

> print(resTogether, quote = FALSE)

> glmMatched <- glm(formula = (V9 == "yes") ~ Group + V1 + V2 + V3 + V5 + V7,

+ family = binomial(link = "logit"),

+ data = matchdata, control=list(maxit=100))

> glmUnmatched <- glm(formula = (V9 == "yes") ~ Group + V1 + V2 + V3 + V5 + V7,

+ family = binomial(link = "logit"),

+ data = mydata, control=list(maxit=100))

> resTogether <- list(Unmatched = ShowRegTable(glmUnmatched, printToggle = FALSE),

+ Matched = ShowRegTable(glmMatched, printToggle = FALSE))

> print(resTogether, quote = FALSE)

> glmMatched <- glm(formula = (V11 == "yes") ~ Group + V1 + V2 + V3 + V5 + V7,

+ family = binomial(link = "logit"),

+ data = matchdata, control=list(maxit=100))

> glmUnmatched <- glm(formula = (V11 == "yes") ~ Group + V1 + V2 + V3 + V5 + V7,

+ family = binomial(link = "logit"),

+ data = mydata, control=list(maxit=100))

> resTogether <- list(Unmatched = ShowRegTable(glmUnmatched, printToggle = FALSE),

+ Matched = ShowRegTable(glmMatched, printToggle = FALSE))

> print(resTogether, quote = FALSE)

> glmMatched <- glm(formula = (V14 == "yes") ~ Group + V1 + V2 + V3 + V5 + V7,

+ family = binomial(link = "logit"),

+ data = matchdata, control=list(maxit=100))

> glmUnmatched <- glm(formula = (V14 == "yes") ~ Group + V1 + V2 + V3 + V5 + V7,

+ family = binomial(link = "logit"),

+ data = mydata, control=list(maxit=100))

> resTogether <- list(Unmatched = ShowRegTable(glmUnmatched, printToggle = FALSE),

+ Matched = ShowRegTable(glmMatched, printToggle = FALSE))

> print(resTogether, quote = FALSE)

> glmMatched <- glm(formula = (V15 == "yes") ~ Group + V1 + V2 + V3 + V5 + V7,

+ family = binomial(link = "logit"),

+ data = matchdata, control=list(maxit=100))

> glmUnmatched <- glm(formula = (V15 == "yes") ~ Group + V1 + V2 + V3 + V5 + V7,

+ family = binomial(link = "logit"),

+ data = mydata, control=list(maxit=100))

> resTogether <- list(Unmatched = ShowRegTable(glmUnmatched, printToggle = FALSE),

+ Matched = ShowRegTable(glmMatched, printToggle = FALSE))

> print(resTogether, quote = FALSE)

> glmMatched <- glm(formula = (V12_0 == "yes") ~ Group + V1 + V2 + V3 + V5 + V7,

+ family = binomial(link = "logit"),

+ data = matchdata, control=list(maxit=100))

> glmUnmatched <- glm(formula = (V12_0 == "yes") ~ Group + V1 + V2 + V3 + V5 + V7,

+ family = binomial(link = "logit"),

+ data = mydata, control=list(maxit=100))

> resTogether <- list(Unmatched = ShowRegTable(glmUnmatched, printToggle = FALSE),

+ Matched = ShowRegTable(glmMatched, printToggle = FALSE))

> print(resTogether, quote = FALSE)

> glmMatched <- glm(formula = (V12_1 == "yes") ~ Group + V1 + V2 + V3 + V5 + V7,

+ family = binomial(link = "logit"),

+ data = matchdata, control=list(maxit=100))

> glmUnmatched <- glm(formula = (V12_1 == "yes") ~ Group + V1 + V2 + V3 + V5 + V7,

+ family = binomial(link = "logit"),

+ data = mydata, control=list(maxit=100))

> resTogether <- list(Unmatched = ShowRegTable(glmUnmatched, printToggle = FALSE),

+ Matched = ShowRegTable(glmMatched, printToggle = FALSE))

> print(resTogether, quote = FALSE)

> glmMatched <- glm(formula = (V12_2 == "yes") ~ Group + V1 + V2 + V3 + V5 + V7,

+ family = binomial(link = "logit"),

+ data = matchdata, control=list(maxit=100))

> glmUnmatched <- glm(formula = (V12_2 == "yes") ~ Group + V1 + V2 + V3 + V5 + V7,

+ family = binomial(link = "logit"),

+ data = mydata, control=list(maxit=100))

> resTogether <- list(Unmatched = ShowRegTable(glmUnmatched, printToggle = FALSE),

+ Matched = ShowRegTable(glmMatched, printToggle = FALSE))

> print(resTogether, quote = FALSE)

> glmMatched <- glm(formula = (V12_3 == "yes") ~ Group + V1 + V2 + V3 + V5 + V7,

+ family = binomial(link = "logit"),

+ data = matchdata, control=list(maxit=100))

> glmUnmatched <- glm(formula = (V12_3 == "yes") ~ Group + V1 + V2 + V3 + V5 + V7,

+ family = binomial(link = "logit"),

+ data = mydata, control=list(maxit=100))

> resTogether <- list(Unmatched = ShowRegTable(glmUnmatched, printToggle = FALSE),

+ Matched = ShowRegTable(glmMatched, printToggle = FALSE))

> print(resTogether, quote = FALSE)

> glmMatched <- glm(formula = (V4_1 == "yes") ~ Group + V1 + V2 + V3 + V5 + V7,

+ family = binomial(link = "logit"),

+ data = matchdata, control=list(maxit=100))

> glmUnmatched <- glm(formula = (V4_1 == "yes") ~ Group + V1 + V2 + V3 + V5 + V7,

+ family = binomial(link = "logit"),

+ data = mydata, control=list(maxit=100))

> resTogether <- list(Unmatched = ShowRegTable(glmUnmatched, printToggle = FALSE),

+ Matched = ShowRegTable(glmMatched, printToggle = FALSE))

> print(resTogether, quote = FALSE)

> glmMatched <- glm(formula = (V_costs == "yes") ~ Group + V1 + V2 + V3 + V5 + V7,

+ family = binomial(link = "logit"),

+ data = matchdata, control=list(maxit=100))

> glmUnmatched <- glm(formula = (V_costs == "yes") ~ Group + V1 + V2 + V3 + V5 + V7,

+ family = binomial(link = "logit"),

+ data = mydata, control=list(maxit=100))

> resTogether <- list(Unmatched = ShowRegTable(glmUnmatched, printToggle = FALSE),

+ Matched = ShowRegTable(glmMatched, printToggle = FALSE))

> print(resTogether, quote = FALSE)

> library(survey)

> library(survminer)

> model <- coxph(Surv(Time,Recurrence) ~ Group + V1 + V2 + V3 + V5 + V7 + V9 + V11 + V13_0 + V14 + V15, id = Number, data = mydata)

> summary(model)

> ggforest(model, data = mydata, main = 'Hazard ratio (95% CI)', cpositions = c(0.10,0.22,0.4),fontsize = 1.0, refLabel = '1', noDigits = 4)
